# The Power of Phase I Studies to Detect Clinical Relevant QTc Prolongation: A Resampling Simulation Study

**DOI:** 10.1155/2015/293564

**Published:** 2015-10-05

**Authors:** Georg Ferber, Ulrike Lorch, Jörg Täubel

**Affiliations:** ^1^Statistik Georg Ferber GmbH, Cagliostrostrasse 14, 4125 Riehen, Switzerland; ^2^Richmond Pharmacology Ltd., St George's, University of London, Cranmer Terrace, London SW17 0RE, UK; ^3^Cardiovascular and Cell Sciences Research Institute, St George's, University of London, Cranmer Terrace, London SW17 0RE, UK

## Abstract

Concentration-effect (CE) models applied to early clinical QT data from healthy subjects are described in the latest E14 Q&A document as promising analysis to characterise QTc prolongation. The challenges faced if one attempts to replace a TQT study by thorough ECG assessments in Phase I based on CE models are the assurance to obtain sufficient power and the establishment of a substitute for the positive control to show assay sensitivity providing protection against false negatives. To demonstrate that CE models in small studies can reliably predict the absence of an effect on QTc, we investigated the role of some key design features in the power of the analysis. Specifically, the form of the CE model, inclusion of subjects on placebo, and sparse sampling on the performance and power of this analysis were investigated. In this study, the simulations conducted by subsampling subjects from 3 different TQT studies showed that CE model with a treatment effect can be used to exclude small QTc effects. The number of placebo subjects was also shown to increase the power to detect an inactive drug preventing false positives while an effect can be underestimated if time points around *t*_max_ are missed.

## 1. Introduction

A specifically designed thorough QT/QTc (TQT) study has been identified by the E14 guideline of the International Conference on Harmonization as a crucial element for clinical assessment of potential cardiac risks of any drug [1]. A dedicated study to determine whether a drug has the potential to prolong the QT interval is conducted in later phases of drug development after proof-of-concept has been established and the pharmacokinetic profile, maximum tolerated dose, and proposed therapeutic dose are determined in Phase I/II studies. The study is solely designed to demonstrate if a drug-induced effect on the heart rate corrected QT interval beyond an upper bound of 10 ms—“the threshold of regulatory concern” [1] can be excluded. This needs to be demonstrated by showing that for each time point the 2-sided 90% confidence interval for the difference of the mean effect and that under time matched placebo are completely below this threshold. As this is the only aim of a TQT study and considering that relatively large sample sizes are required for the study, the cost-effectiveness of this type of approach has been discussed [2]. An important component of a TQT study is the use of an active control to demonstrate the sensitivity of the assay [3]. Moxifloxacin is commonly used in this role and the use of this antibacterial fluoroquinolone outside its indication has contributed to a search for alternatives to conventional TQT studies [4].

It has been conjectured that, without compromising the QT assessment, increased efficiency can be attained by collecting the same quality QT data in single ascending dose (SAD) and multiple ascending dose (MAD) first in human studies. Cardiac safety assessment is not the primary objective of these early studies but as these studies often use doses up to the maximum tolerated dose (MTD) achieving plasma concentrations above those that will be seen during later stages of development, SAD and MAD studies are the ideal candidates for incorporation of early QT assessment [5–8].

This search for alternatives to a TQT study and the use of QTc data obtained in Phase I studies have been extensively discussed [9]. One question of outstanding interest is whether analyses based on data obtained from these studies will have a sufficient power to reliably show QTc prolongation and, more importantly, to reliably predict the absence of such an effect. Substantial differences between a TQT study and a SAD or MAD study must be considered. Although the total number of subjects involved in a SAD study may not be much less than in a crossover TQT study, only a fraction of them are exposed to drug doses that are at or above the level that will be used in future therapies. Moreover, while in a TQT study one proposed therapeutic dose and one supratherapeutic dose of the drug are used, in a SAD or MAD study several doses are employed and only a few subjects are given each of the doses. Furthermore, ECG monitoring in SAD/MAD studies may be limited as these studies are primarily designed to address subject safety and to exclude only large electrocardiographic abnormalities which implies a modification of these Phase I studies to integrate robust ECG monitoring and analyses.

Concentration-effect (CE) modelling is a well-established method already used as a secondary analysis in TQT studies [10]. The appropriateness of a CE analysis based on the change from baseline was shown to be a valid alternative recognizing that model selection can be improved with experience and more analysis of data from drugs with a known effect on QTc can help to substantiate the model [11].

Even though the potential of applying CE analysis to QTc data generated from SAD and MAD studies has been recognised, the level of confidence and the power of such an analysis in a situation such as a Phase I study are still one of the key points to be addressed. Ferber et al. [11] used subsampling from crossover TQT study data to simulate small studies and showed that sample sizes of 9 subjects on active drug and 6 on placebo provide sufficient power to detect or exclude an effect similar to the one of moxifloxacin. In this publication, we replicate these findings based on a different set of TQT studies and, in addition, we investigate the role of design features in the power of the analysis. In other words, we attempt to broaden the understanding of the power of CE analysis for detecting clinically significant QTc prolongation and exclude such an effect for an inactive drug.

## 2. Methods

The simulation work was based on data from three crossover TQT studies in healthy volunteers. In these studies, a single dose of 400 mg of moxifloxacin was given as a positive control. Data from subjects with available ECG and PK data from moxifloxacin and placebo treatment were used.

*Study 1.* This randomised, placebo-controlled, double blind crossover study consisted of 96 volunteers. Moxifloxacin was given in the fasting state on day 16 of the moxifloxacin study period (placebo given on 15 preceding days). ECG data were collected on day 16 of the moxifloxacin period at 12 time points: predose, 0.5, 1, 1.5, 2, 3, 4, 5, 6, 8, 12, and 24 h postdose [12]. 

*Study 2.* This randomised, placebo-controlled, double blind crossover study consisted of 64 volunteers. Moxifloxacin was administered in the fasting state on day 2 of the moxifloxacin study period (placebo given on the preceding day). ECG data were collected at 12 time points: predose, 0.5, 1, 1.5, 2, 2.5, 3, 3.5, 4, 8, 12, and 24 h postdose [13]. 

*Study 3.* This randomised, placebo-controlled, double blind crossover study consisted of 49 volunteers. Moxifloxacin was given in the fasting state on day 1 of the moxifloxacin study period, preceded by placebo on a baseline day. ECG data were collected at 14 time points: predose, 0.5, 1, 1.5, 2, 2.5, 3, 4, 5, 6, 8, 10, 12, and 24 h postdose [14].

For all studies, 12-lead ECGs were recorded and stored electronically on the MUSE CV information system (GE Healthcare). Before any ECG recording, the subjects maintained an undisturbed supine resting position for at least 10 minutes and avoided postural changes during the ECG recordings. At each time point, the ECGs were recorded in triplicate at 1-minute intervals during 3 minutes. Each ECG lasted 10 seconds.

Automatic ECG analysis was performed by the Marquette 12SL ECG Analysis Program (MEAP). All ECGs and their associated automated interval measurements were subsequently reviewed by qualified cardiologists. If manual adjustments of the automated measurement became necessary, a second cardiologist confirmed the assessment. Any disagreement between first and second readers was adjudicated by a third and most senior cardiologist. Details of this process have been described in [15]. For further analysis, the mean across the triplicates was used.

In our simulation studies, we used QT corrected according to Fridericia (QTcF) [16]. In particular, we did not consider subject-individual corrections, which may contribute to an undue complexity of a Phase I study and may be unnecessary in the presence of small heart rate effects.

### 2.1. Data Analysis

The analysis method used has been described elsewhere [11]. By taking a subsample of subjects, data under placebo and under active drug (moxifloxacin) can be obtained. To simulate a drug that does not prolong QTc, PK data obtained under moxifloxacin was combined with the time matched QTcF values from the same subjects under placebo. Data from all time points or only data from a subset of time points were used.

Each simulated study was assessed for a QT-prolongation of regulatory concern using a concentration-effect modelling approach according to the methods described in [9]. It was considered negative if the two-sided 90% confidence interval for the effect predicted at the geometric mean *C*_max_ was completely below 10 ms. More specifically, two concentration-effect models were fitted to each simulated study as follows:

 
*Fixed effects*:
  (1) ΔQTcF~C+time+teatmentΔQTcF~C+time
 
*Random effects*: intercept per subject.

The models use the change from predose baseline of QTcF as dependent variable and concentration as a covariate. In order to correct for spontaneous circadian effects, a factor representing time was also added [17]. The two models differ in the inclusion of an additional treatment effect not forcing the slope through the origin (zero) [11]. Only an intercept per subject was included as a random effect. From each model, the effect at the observed geometric mean *C*_max_ was predicted together with a two-sided 90% confidence interval. The random variability of the *C*_max_ estimate was not taken into account in order to keep the computational burden within reasonable limits. A study was declared negative if the upper bound of the confidence interval was below the threshold of 10 ms, as per ICH E14 guideline [1].

One thousand simulations were performed for each configuration and each TQT study by sampling *N*_act_ + *N*_pla_ subjects without replacement, where *N*_act_ and *N*_pla_ are the number of subjects used under active drug and under placebo, respectively. The fraction of negative studies out of these simulations was determined. For the method to produce reliable results, this fraction of negative studies should be below a threshold of 5% for an active drug like moxifloxacin, while for an inactive drug, at least 85 or 90% of the simulated studies should be negative.

In a first step, data from simulations with the same number of subjects on active drug and on placebo was used and 6, 9, 12, 15, and 18 subjects per group were selected. The fraction of negative studies was displayed for the two types of models and for moxifloxacin and the simulated inactive drug.

To investigate the role of subjects under placebo in the CE analysis, simulations were conducted by fixing the number of subjects on active drug to 9 and varying the number of subjects on placebo from 3 to 6 and 9. Results for the model with a treatment effect were given for moxifloxacin and for the simulated inactive drug.

Finally, the number of time points included in the models was reduced to investigate the influence of the number of time points in general and the importance of sampling around *C*_max_. Therefore, the time points were subsampled according to the scenarios depicted in Table 1. This investigation was performed for a scenario with 9 subjects on drug and 6 on placebo and was based on a model with a treatment effect. The fraction of false negatives was displayed for moxifloxacin and the fraction of false positives for the simulated inactive drug. All computations were performed using *R* [18] and in particular the package nlme [19].

## 3. Results

**Figure 1:**
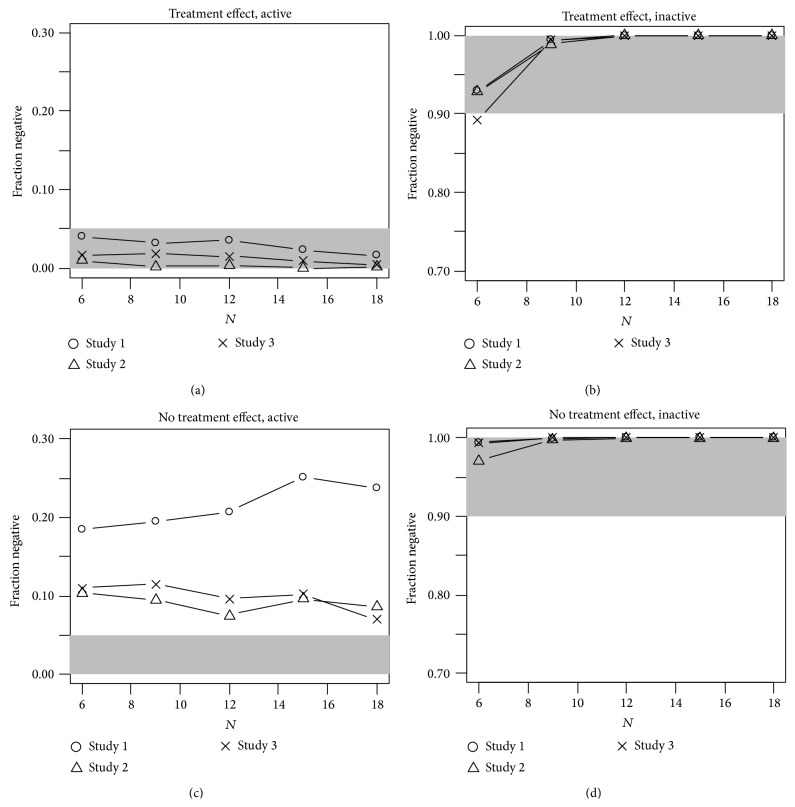
Fraction of negative studies by number of subjects per treatment arm. (a) and (b) Analysis with a model with a treatment effect; (c) and (d) analysis with a model without a treatment effect. Shaded range is considered acceptable.

**Figure 2:**
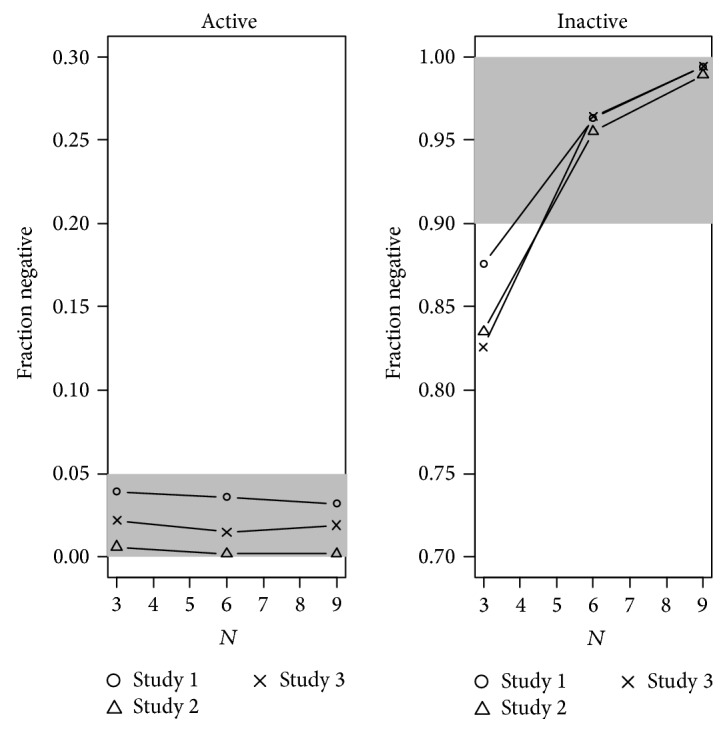
Power of CE modeling as a function of the number of subjects on placebo. In all simulations, 9 subjects were used in the active group, while the number on placebo was varied as given on the *x*-axis. Shaded range is considered acceptable.

Using the model with treatment effect, the fraction of negative studies as function of the sample size (per treatment group) is displayed in Figures 1 and 2.  Figure 1(b) shows that the CE method using a model with treatment effect reliably excludes an effect in an inactive drug, while Figure 1(a) shows that it detects an effect, such as the one caused by moxifloxacin. The rate of false negatives is below 5% for all studies and all sample sizes considered (Figure 1(a)), and studies based on the simulated inactive drug are correctly classified as negative in more than 95% of the case for sample sizes of *N* ≥ 9.

When a concentration-effect model without a treatment effect is used, a clear effect on the fraction of negative studies becomes apparent.  Figure 1(c) shows that with a model without a treatment effect the fraction of negative studies was never below 5% for all studies while with a model with treatment effect it was below 5% for all cases. In the “no-effect scenario,” the fraction of negative studies was higher with a model without treatment effect, above 95% even with 6 subjects (Figure 1(d)).

Figure 2 gives the fraction of negative studies with 9 subjects on active drug as a function of the number of subjects on placebo. It shows that, using the model with a treatment effect and only 3 subjects on placebo, a “moxi-like” effect can reliably be detected, while a larger number of subjects on placebo improve the power to exclude a QT-prolonging effect in an inactive drug from above 80% to >95%. With 9 subjects on active drug and 6 subjects on placebo, the fraction of false positive studies can be reduced below 5%, providing a power of >95%.

**Figure 3:**
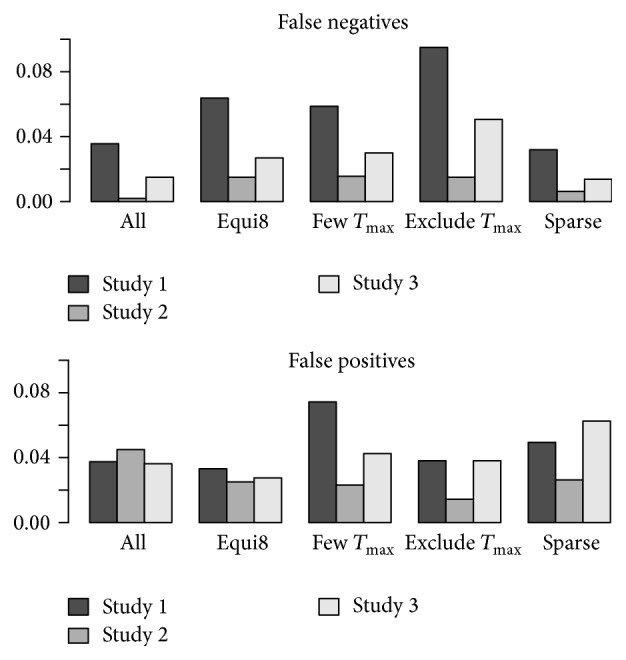
Performance of CE analysis as a function of the time points included. Simulations were based on model with treatment effect with 9 subjects on active drug and 6 on placebo.

The results of simulations based on a reduced number of time points are given in Figure 3. Overall, the reduction of the number of time points does not seem to have a strong influence on the performance of the method when the scenario “All” is compared with the remaining scenarios. However, reducing the number of time points around *t*_max_ results in a slight increase of false negative studies, as represented by the “Exclude *t*_max_” scenario. This increase of false negatives is apparent in the results from Study 1 and, to some extent, by Study 3, but not suggested by Study 2. The influence of the selection of time points on the fraction of false positives is not very pronounced.

## 4. Discussion

In this paper, we used real data from 3 published TQT studies to confirm the findings from Ferber et al. [11], based on another set of studies and, in addition, we investigate the dependence of the results on the selection of time points and on the role of placebo subjects for excluding an effect for an inactive drug. With this approach, we intend to broaden the basis of knowledge on the applicability and the behaviour of CE modelling in small studies.

In order to be acceptable to regulators as an alternative to a TQT study, the rate of false negatives in the analysis of QTc data from SAD or MAD studies must be low. On the other hand, if the method has a substantial risk for a false positive result with respect to QTc prolongation, it becomes unattractive for the sponsor, since, as a minimum, an additional TQT study has to be performed in such a case. Therefore, a good control of the false positive rate is in the interest of the sponsor.

The subsampling simulations presented here confirm that with 9 subjects on active drug and 6 on placebo, the fraction of false negatives under moxifloxacin and that of false positives under a simulated drug with no effect on QT and the pharmacokinetic properties of moxifloxacin are well controlled. All studies presented a rate of false negatives and false positives, respectively, below 5% (Figure 2). These sample sizes are close to or below what is usually achieved in the highest dose groups of a SAD study and in the pooled placebo.

The importance of the treatment effect in the model to control the fraction of false negatives has been observed on different data [11]. In terms of the rate of false negatives, when treatment effect is omitted in the CE model and the linear regression is not forced to pass through zero, a rate increase is observed (Figure 1(c)) while the likelihood of false positive results is lower (Figure 1(d)). At this point in time, one can only speculate about the reasons for this phenomenon. A significant treatment effect is usually taken as a sign that model fit can be improved by taking into account nonlinearity and/or hysteresis. However, it should be kept in mind that the goal of CE analysis in Phase I studies is the reliable detection of a QT effect of regulatory concern and not an explanatory description of the PK-PD relationship. Therefore, using a model with treatment effect as default seems to be a reasonable choice. In a real Phase I study, a significant treatment effect would probably trigger further investigations into the appropriateness of the model used. The prospective definition of criteria to ascertain model fit with respect to a greater delay between pharmacokinetics and pharmacodynamics as well as to linearity is a key feature of CE analysis in Phase I studies and one of the topics of current research [9].

The results on the number of subjects on placebo included reinforcing the importance of placebo to obtain a reliable prediction. It may be speculated that the subjects on placebo provide the basis for a reliable estimate of the spontaneous variability over time and allow discriminating this from a drug-mediated effect if the time course of drug concentration is similar to the spontaneous changes.

Another feature varied in this study was the creation of different scenarios with selected time points to determine the importance of the time points around *C*_max_. Bearing in mind that in a TQT study the estimated QTc effect at the highest clinically relevant plasma concentration will define the QTc effect of a drug, the appropriate selection of time points around *C*_max_ is important to predict the maximum effect. Surprisingly, the method seems relatively stable against the selection of time points. However, a closer look at the characteristics of the three studies explains this finding.

**Figure 4:**
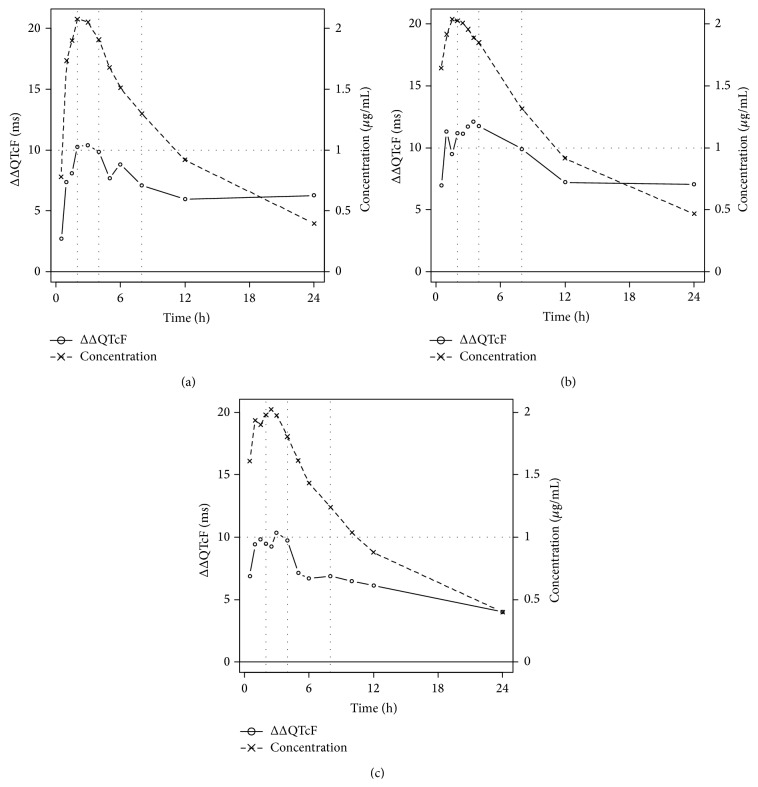
Time course of moxifloxacin plasma concentration and placebo corrected change from baseline of QTcF for each of the three studies, based on all subjects included. (a) Study 1, (b) Study 2, and (c) Study 3.

Figure 4 presents the mean time course of the plasma concentration of moxifloxacin as well as the placebo corrected change from baseline of QTcF (ΔΔQTcF) based on all subjects in the respective TQT study. As demonstrated, in all three studies the rise of plasma concentrations and of placebo corrected QTcF starts early and values close to *C*_max_ are already reached in the first two hours. In particular, *t*_max_ for Study 2 is well before 2 h (Figure 4(b)). Excluding values in the time window 2–4 h therefore will not remove high concentrations from the model in Study 2 and an effect on the number of false positives is not observed. Only for Study 1 the concentrations outside the window 2–4 h are clearly lower than those in this window. Accordingly, this study shows the highest number of false negatives under the “Exclude *t*_max_” scenario, indicating that if *t*_max_ is missed, the effect will be underestimated.

On the other hand, even reducing the number of time points to 4 per subject (Sparse scenario) seems to have little influence on the quality of the model fit as can be judged from the fraction of misclassifications presented here (Figure 3).

A limitation of this investigation is that all simulations are based on moxifloxacin and, furthermore, 400 mg is not a supratherapeutic dose. Similar investigations using drugs with other kinetics or with a more complex PK/PD relationship would therefore be useful.

In early phase studies, the doses investigated are usually higher than all doses used in later phases and more than one cohort will contribute to the analysis. As a criterion for a negative QT assessment based on SAD data for CE analysis is to evaluate the QTc effect at plasma concentrations that cover levels seen in patients with impaired clearance and high plasma concentrations of the drug [6], it seems reasonable to expect that, using data from a SAD study, a QTc effect above 10 ms can be excluded with a likelihood of less than 5%.

Also important to note is that this study, as well as other similar simulations studies [9, 11], is based on data from traditionally designed TQT studies primarily focused on ECG analysis. Phase I studies are mainly focused in the pharmacokinetic and safety assessments and often include pharmacodynamics assessments which all can interfere with the accuracy of ECGs measurements. Additionally, eventual adverse events caused by high doses and general nervousness surrounding a first-in-human drug administration can affect the autonomic responses altering the QT/RR relationship which can also present a limitation for the use of Phase I data in simulation studies to detect clinical relevant QTc prolongation.

CE modelling with varied number of subjects on placebo was carried out and showed the importance of placebo for the power of the method, that is, the ability to reliably exclude an effect in an inactive drug. Placebo is routinely included in Phase I studies and, therefore, obtaining a sufficient number of subjects under placebo is not considered an issue.

The apparent robustness of the method against misplacement of time points—as long as values near the maximum concentration are not completely absent—is reassuring. However, at least until a more in depth understanding of the method is reached, the absence of a positive control remains an issue. SAD and MAD studies do not typically include a pharmacological control to confirm ECG assay sensitivity. This is considered a major limitation when using their data to exclude an effect as systematic errors may have occurred limiting the sensitivity of a study thereby giving a false negative result. Unlike random error, which will lead to wider confidence intervals and thereby will not allow excluding a 10 ms change in QTc, systematic errors cannot be reliably detected other than by including a positive control.

Several paths to overcome this lack are being investigated, but up to now, no solution has been generally accepted. We have demonstrated previously that one to four hours after the intake of a carbohydrate rich meal, a physiological QTc-shortening can be reliably observed [4]. The change in QTc is closely correlated with the release of c-peptide in response to raising blood glucose concentrations after a meal and thus is a physiological response rather than the effect of a blocking drug that may be differently metabolised in a significant proportion of the study population [20].

In the setting of a Phase I study, this effect can be estimated from the time course of the change from baseline of QTc that is a byproduct of the CE analysis. We therefore suggest that assessing this shortening of QT by about 5 ms 2–4 h after meal intake compared to the predose value can be developed into a proof of assay sensitivity [21]. It should be noted that the proposed test for assay sensitivity is based on the same data as the investigation of the drug effect on QTcF and is estimated by the same model as the primary analysis [17]. In this way, it becomes unlikely that a systematic error acts only on the drug effect, but not on that of food.

## 5. Conclusions

This study describes the power of simulated small studies and their use towards the acceptance of alternative approaches that can provide data at the same level of confidence of a TQT study. Here, we focused on a suitable analysis method for the setting of a Phase I study.

Our approach was based on the performance of CE analysis to investigate the confidence and the power of the analysis for detection of QTc changes of clinical relevance by subsampling subjects from three different TQT studies. This simulation confirmed that QTc prolongation can be reliably detected with a small sample size and a drug not causing any QTc prolongation can be identified with a power of more than 90%. Additionally, the power to detect an inactive drug is increased with the number of subjects on placebo, with 6 subjects on placebo showing good power. Although it is important to have a sufficient number of PK and QTc samples around *t*_max_ to avoid false negative findings, the simulations underline the robustness of the model even if the number of time points is reduced. The choice of an adequate statistical model that includes a treatment effect seems to be important to fully exploit the potential of this method. Our study supports that a model with a treatment effect can be used to exclude a QTc effect similar to moxifloxacin as the fraction of negatives studies is below 5%. However, the influence of pharmacokinetic parameters on the rate of false positives should be further explored to evaluate how lower doses can influence the precision of the concentration/QTc effect model to be used in QT assessment studies.

The proposal to use the effect of food as positive control fits well with the use of a CE model as it can be based on the same data. The results therefore support the assumption that, in many cases, this methodology, based on high quality ECG data, could be used instead of the TQT study that constitutes significant financial burden on clinical stages of drug development.

## Figures and Tables

**Table 1:** Scenarios used to investigate the influence of selection of time points.

Designation	Maximum total number of time points	Number of time points in time window			
		0 < *t* < 2 h	2 h ≤ *t* ≤ 4 h	4 h < *t* ≤ 8 h	8 h < *t* ≤ 24 h
All	All	All	All	All	All
Equi 8	8	At most 2	At most 2	At most 2	At most 2
Few *t*_max⁡_	7	At most 2	At most 1	At most 2	At most 2
Exclude *t*_max⁡_	6	At most 2	None	At most 2	At most 2
Sparse	4	At most 1	At most 1	At most 1	At most 1
